# Structure, optical properties, TD-DFT simulations for nanosecond and continuous laser irradiation of vanadium antimony borate glass doped with nickel ferrite

**DOI:** 10.1038/s41598-023-50364-1

**Published:** 2024-01-02

**Authors:** A. R. Ghazy, S. A. Abdel Gawad, R. Ghazy, A. N. EL‑Sharkawy, O. M. Hemeda, A. M. A. Henaish

**Affiliations:** 1 Physics Department, Faculty of Science, Tanta University, Tanta, 31527 Egypt; 2 Basic Science Center, Misr University for Science and Technology (MUST), 6 of October, Egypt; 3 NANOTECH Center, Ural Federal University, Yekaterinburg, Russia 620002

**Keywords:** Applied physics, Optical physics

## Abstract

Borate antimony glass doped with vanadium oxide V_2_O_5_ encoded into a [BSV glass system] was prepared with the traditional melt quenching technique. The Nickel ferrite [NiFe_2_O_4_] was prepared using Flash auto-combustion and mixed at a fixed ratio of 0.05 gm into the glass matrix to form a BSV- composite glass system [BSV / NiFe_2_O_4_], which was also prepared using the traditional melt quenching technique. The X-Ray diffraction pattern was used to characterize the glass samples and indicated their amorphous structure, with different structure phases for different levels of V_2_O_5_ content. Ranging from 200 to 1100 nm, UV–Vis spectroscopy was used to study the optical properties of the samples. The glass was found to absorb electromagnetic waves with wavelengths lower than 500 nm, while the energy gap decreased from 2.46 eV for 0.1 mol% V_2_O_5_ to 2.39 eV for 0.5 wt% V_2_O_5_. The Urbach energy also had the same behavior, and decreasing from 0.226 to 0.217 eV. On the other hand, the refractive index increased when V_2_O_5_ was added. The thermal characteristics of a [BSV / NiFe_2_O_4_] system, such as, glass transition temperature ${T}_{G}$, onset temperature ${T}_{X}$, crystallization temperature ${T}_{C}$ and melting temperature ${T}_{m},$ were studied using a Differential Scanning Calorimeter. Using continuous and pulsed laser radiation, a [BSV-0.1 V_2_O_5_ / NiFe_2_O_4_] sample was exposed to laser irradiation to observe its effect on the optical features of the glass. Laser irradiation significantly changed the absorbance spectrum, while the energy gap decreased as time increased. The pulsed laser was found to have a more power-full and uniform effect compared to continuous laser. Time-dependent density function theory was used to optimize the geometrical structure of the glass and study the effect of the formation of 4- coordinate boron atoms on its properties.

## Introduction

In recent years, scientists have paid attention to borate glasses due to their impressive properties when compared to silicate glasses^1^. Chemical sturdiness, low melting temperature, thermal stability and transparency are the properties that make borate glasses useful in in solid state electronic equipment, such as liquid–crystal displays, light emitting diodes and touch screens^2^. Other characteristics of boron glasses include reliability, long lifespan, high-power energy and low carbon emissions^3–5^.

Nickle ferrite is a spinel ferrite with a structural formula of NiFe_2_O_4_. It is known as a soft magnetic material and is used in many applications such as microwave devices^6–8^, photoelectric devices^9^, magnetic pigments^10^, nano devices^11^, sensors^12^ and catalysis^13^. This is due to its impressive properties such as low magnetic moment and saturation temperature, high expansion coefficient, high specific heat and low melting point^14,15^.

Vanadium oxide is a transition metal that can be used in a wide range of applications such as radiation shielding, optoelectronics and solar cells^16,17^. Doping in borate glasses with vanadium oxide improves the field strength, ion size and glass structure, making the glass a semiconductor^18–21^. As a result, vanadium doped glasses are used in optoelectronics, gamma radiation shielding and memory switching applications^22,23^. Since the state of the vanadium ions and the number of defects influence the properties of the glass, vanadium doped glass has been mainly studied with high concentrations of vanadium oxide; there is a lack of studies of glass doped with low concentrations of vanadium oxide.

In recent years, estimating the spectroscopic properties of different molecular structures using complete energy-based simulation methods has received much attention. Time dependent density function theory (TD-DFT) is the basis of these calculations, providing different simulation techniques such as the DMol^3^ and CASTEP techniques^24,25^. A limited programming language is used to apply potential energy of HUMO and LUMO states and geometrical studies^26^. A high level of precision is achieved by consistently employing same atomistic modeling techniques^27^. When the electron–ion potential is presented by using ab initio pseudopotentials during ultrasoft and standard-memorizing formulations and the conscience-consistent method, Kohn–Sham wave functions and charge relevant intensity are derived depending on direct energy reduction. Strong DFT electron can be used to represent the system shape with a finite inhabitant number^28,29^. Measurement convergence for different k-point composite compounds is influenced by the base set size supplied by plane waves cut-off and precise integration of the Brillouin zone^30^.

Laser irradiation of solid materials has attracted much attention recently due to its ability to influence the structural, optical and electrical properties of irradiated material. Laser irradiation has advantages over traditional furnace annealing such as increased charge carriers, decreased thermal exposure and rapid room temperature crystallization^31–34^. Absorption, transmission, scattering and reflection can be used to characterize the laser irradiation process. Photon absorption can be considered the most effective, since it can alter a material’s chemical and physical properties^35,36^.

Spinel ferrite as a dopant has attracted the researcher’s attention in recent years due to its applications in gamma attenuation and radiation shielding. Hannachi et al.^37^ studied the radiation shielding of a series of ceramic composites doped with spinel ferrites and found that the presence of spinel ferrites improved the shielding performance of the composites. Alsaif et al.^38^ studied the radiation shielding properties of polyvinyl chloride doped with nickel lead ferrite and recommended the composite to be used in radiation shielding. On the other hand, many researchers have worked on the laser irradiation of glass. Liu et al.^39^ used femtosecond laser irradiation to micromodificate element distribution in the glass matrix. Zhang et al.^40^ improved the surface hardness of the glass using nanosecond pulsed laser irradiation. Menazea et al.^41,42^ used nanosecond laser irradiation to participate silver nanoparticles in both borate and silicate glasses. The current study is original in that it will investigate the influence of laser irradiation on vanadium doped nickel ferrite borate antimony glass. It will use continuous and nanosecond pulsed lasers and study the differences between them in terms of their effect on optical properties.

In this work, in order to obtain a high optical absorbance glass material with good mechanical strength, vanadium borate antimony glass doped with nickel ferrite [BSV / NiFe_2_O_4_] was prepared by the traditional melt quenching technique. The structural, optical, and thermal features of the synthesized samples were studied. TD-DFT simulations were performed on an isolated [BSV / NiFe_2_O_4_]^Iso^ molecule to study the effect of 4-coordinate boron atoms on the properties of the glass, along with structural and optical studies. Finally, a [BSV-0.1 V_2_O_5_ / NiFe_2_O_4_] sample was exposed to continuous and nano second pulsed laser irradiation over different irradiation time and the change in the optical properties was studied.

## Experimental details

### Sample preparation

**Figure 1:**
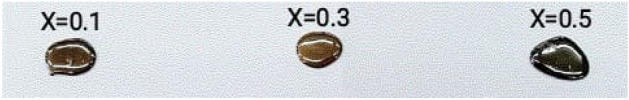
Molded [BSV / NiFe_2_O_4_].

Borate antimony glass doped with vanadium oxide V_2_O_5_ and nickel ferrite NiFe_2_O_4_ was prepared with a composition of (49.95-x) B_2_O_3_ + 50 Sb_2_O_3_ + 0.05 NiFe_2_O_4_ + x V_2_O_5_, x = (0.1, 0.3 and 0.5 wt%) using traditional melt quenching technique^43^. The weighted precursors were fully mixed to get a uniform compositional mixture and finely ground in porcelain crucibles, which were kept inside an electric furnace for melting. The glass blends were maintained at a temperature of 800 °C for about 20 min. The prepared glasses were molded at 150 °C using a stainless-steel mold to take the shape of discs, as shown in Fig. 1.

### Characterization methods and techniques

The amorphous character of the glass samples was confirmed using The X-ray diffraction (XRD) technique with a Cu K_α_ radiation source (λ = 1.54 nm) (Philips model—PW-1729, Germany) step size 0.02 °C; time per step: 21 s. On the Jasco FT/IR-4000 spectrometer (Japan), KBr pellet infrared spectra were captured. The UV/visible spectrum of the glass when using a Jasco V-630 UV–Vis spectrophotometer (Japan) with a double-beam has a single monochromator covering wavelength range of 190–1100 nm with a fixed bandpass of 1.5 nm and scanning speed up to 8,000 nm/min. The differential scanning calorimeter (DSC) was set with a Setaram LABSYS evo DSC (France). The emission spectra were measured from a laser photoluminescence experiment with a 325 nm He-Cd laser at 150 mW of power. The HoRiBA, Japan (IHR 320) spectrum analyzer with a computerized CCD camera was used to record the emission spectra. The influence of laser radiation was tested using a diode continuous laser (375 nm wavelength 150 mW power) and a pulsed laser (1064 nm wavelength and 50 mW power nanosecond pulse duration).

## Results and discussion

### Fourier Transform Infrared Spectroscopy (FTIR)

**Figure 2:**
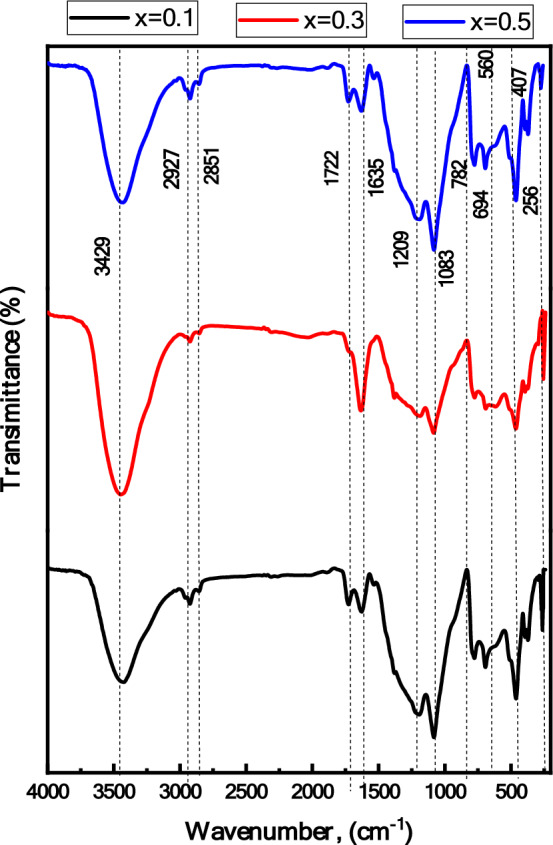
FTIR spectra of [BSV / NiFe_2_O_4_] glass composite.

The FTIR spectra of BSV glass composite doped with ferrite are shown in Fig. 2. For different V2O5 and constant ratio of nickel ferrite. The spectra show similar functional group as the BSV glass system in addition to the two characteristic bands appeared at far infrared region which assigned to octahedral and tetrahedral group complex for Fe^3+^_O^2-^. The absorption bands that appear in Fig. 2 are summarized in Table 1.

**Table 1:** The absorption band positions obtained from FTIR spectra.

Sample	0.1	0.3	0.5	Assignment	References
A	368	368	368	Octahedral absorption band of Fe^3+_^ O^2-^	^44,45^
B	456	469	456	Vibration of Fe–O bond in tetrahedral site	^44,46,47^
C	694	694	694	Bending vibration B–O–B bonds and Vs=O bond	^19,44^
D	782	782	782	Stretching vibration of B_O bond in Bo_4_ group	^44,45,48^
E	1083	1070	1083	V=O double bond	^19^
F	1209	1209	1209	symmetric stretching in Bo_3_ units from pyro and Orth-borate groups and Bo_4_ group	^19,47^
G	–	1396	–	Asymmetric stretching of B–O of trigonal Bo_3_	^45,49^
H	1635	1635	1635	Combination of relaxation of B_O band of trigonal Bo_3_ units in glass matrix and C=O in ferrite	^50^
I	2851	2851	2851	Hydrogen bonding	^51^
J	2927	2927	2927	Hydrogen bonding	^44^
K	3453	3440	3429	Molecular water	^44^

### XRD of BSV /Ferrite glass composite

**Figure 3:**
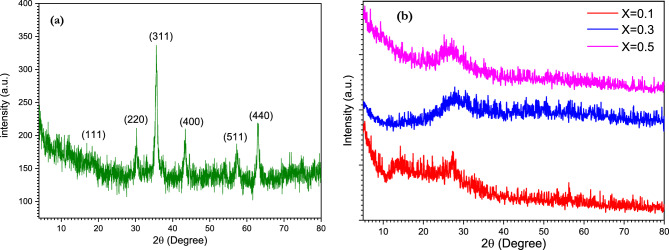
XRD of (**a**) NiFe_2_O_4_ and (**b**) [BSV / NiFe_2_O_4_] glass composite.

The XRD analysis in this article focuses on the crystallization and amorphous nature of the glass samples. The XRD pattern of NiFe_2_O_4_ and [BSV / NiFe_2_O_4_] are shown in Fig. 3a and b. As shown in Fig. 3a the cubic spinel structure of Nickel ferrite is confirmed by the appearance of (111), (220), (311), (400), (511) and (440) diffraction peaks. The values of lattice parameter and crystallite size of NiFe_2_O_4_ were found to be 0.843 and 25 nm respectively which were found to match the literature values of the nickel ferrite^52^. The XRD analyses of [BSV / NiFe_2_O_4_] with different concentrations of V_2_O_5_ are shown in Fig. 3b. The amorphous nature of the glass is confirmed by the XRD patterns in Fig. 3. The XRD pattern exhibits broad diffusion at 2θ = 27°, which indicates the presence of long-range structure disorder and confirms an amorphous nature^53,54^. XRD confirms the amorphous nature of glass, even though ferrite is present in the network^55^. B_2_O_3_ and V_2_O_5_ are good modifiers: the presence of both supports the amorphous nature of the BSV/ ferrite composite^56^.

**Figure 4:**
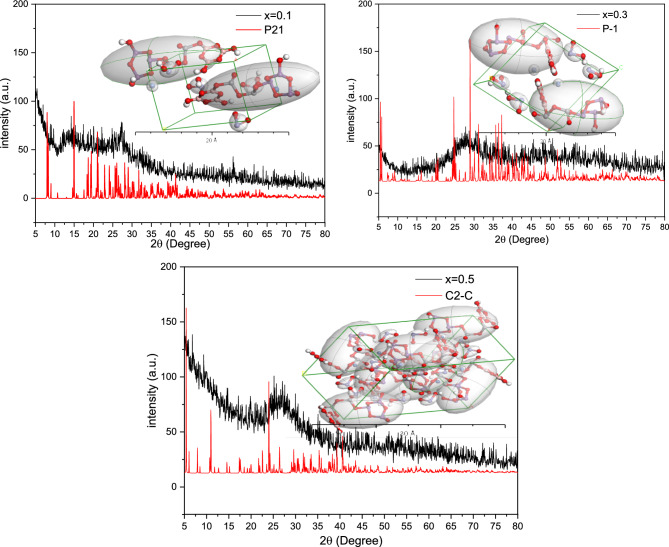
Comparing the experimental XRD data with polymorph simulated data for different glass compositions.

By applying a polymorph calculation module, different X-ray diffraction models were computed for the glass samples. By comparing the experimental XRD data with the computed models, it can be demonstrated that the samples have different structures depending on the V_2_O_5_ content. Figure 4a, b and c shows the experimental XRD patterns with the corresponding theoretical data, where the inset figures represent the 2 × 2 × 1 Brillouin zones and performed integrals. For 0.1 V_2_O_5_, the glass had a *monoclinic* P21 phase with lattice parameters of a = 15.7Ǻ, b = 8.3Ǻ, c = 9.2Ǻ, α = γ = 90° and β = 103.9°. When the V_2_O_5_ content was increased to 0.3 the structure of the glass changed to *triclinic* P-1 phase with lattice parameters of a = 8.3Ǻ, b = 12.5Ǻ, c = 11.4Ǻ, α = 103.4, β = 103.8° and γ = 78.5°. Finally, for 0.5 V_2_O_5_, the structure turned into a *monoclinic* C2-C (phase a = 25.2Ǻ, b = 12.9Ǻ, c = 14.4Ǻ, α = γ = 90° and β = 82.5°).

### DSC of BSV / ferrite composite

**Figure 5:**
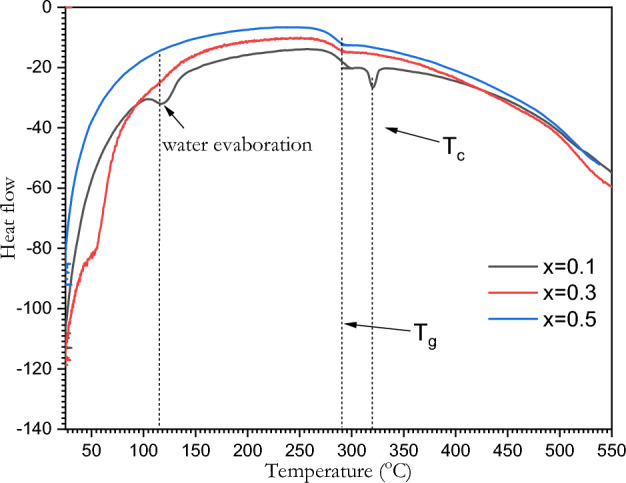
DSC of the [BSV / NiFe_2_O_4_] glass system.

A differential scanning calorimeter (DSC) was used to investigate the thermal characteristics of ([BSV / NiFe_2_O_4_] as shown in Fig. 5. It can be figured out that water molecules were evaporated at temperature range of 113–116 °C. while the glass transition temperature T_g_ ranged between 276 and 296 °C. Finally, the crystalline temperature T_c_ found to be range between 285 and 319 °C.

### TD-DFT simulations of an isolated borate antimony glass molecule

**Figure 6:**
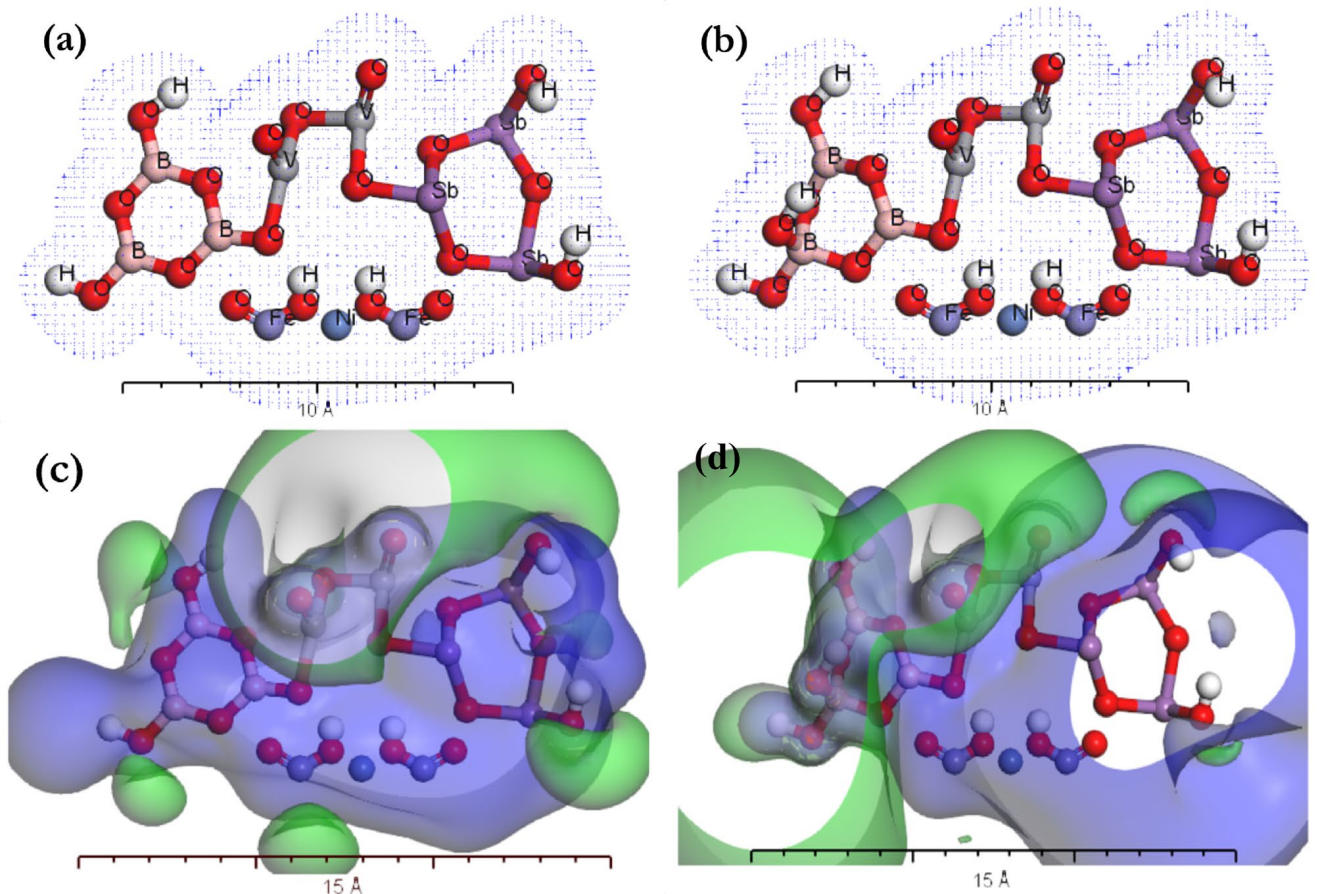
(**a** and** b**) electron density, (**c** and** d**) electrostatic potential for broxol and modified boroxol rings in [BSV / NiFe_2_O_4_]^Iso^.

In order to study the effect of the formation of 4-coordinate boron atoms, the similar properties of the gaseous phase of borate antimony glass [BSV / NiFe_2_O_4_]^Iso^ were studied for boroxol and modified boroxol rings using electron density and electrostatic potential (see Fig. 6)^57,58^. The geometrical optimization of [BSV / NiFe_2_O_4_]^Iso^ was achieved by applying the TD-DFT/ *Dmol*^*3*^ simulation model using the Perdew Wang function (LDA/PWC). The gaseous phase electron systems of [BSV / NiFe_2_O_4_]^Iso^ as isolated molecules were studied using TD-DFT simulations as the electron density (see Fig. 6a and b). As in Fig. 6c and d the potential growth of the [BSV / NiFe_2_O_4_]^Iso^ gas phase was investigated. As a result, the electron transfer possibilities in the gas phase of [BSV / NiFe_2_O_4_]^Iso^ were supported. The 4-coordinate boron atom has an obvious effect on electron density and electrostatic potential maps.

**Figure 7:**
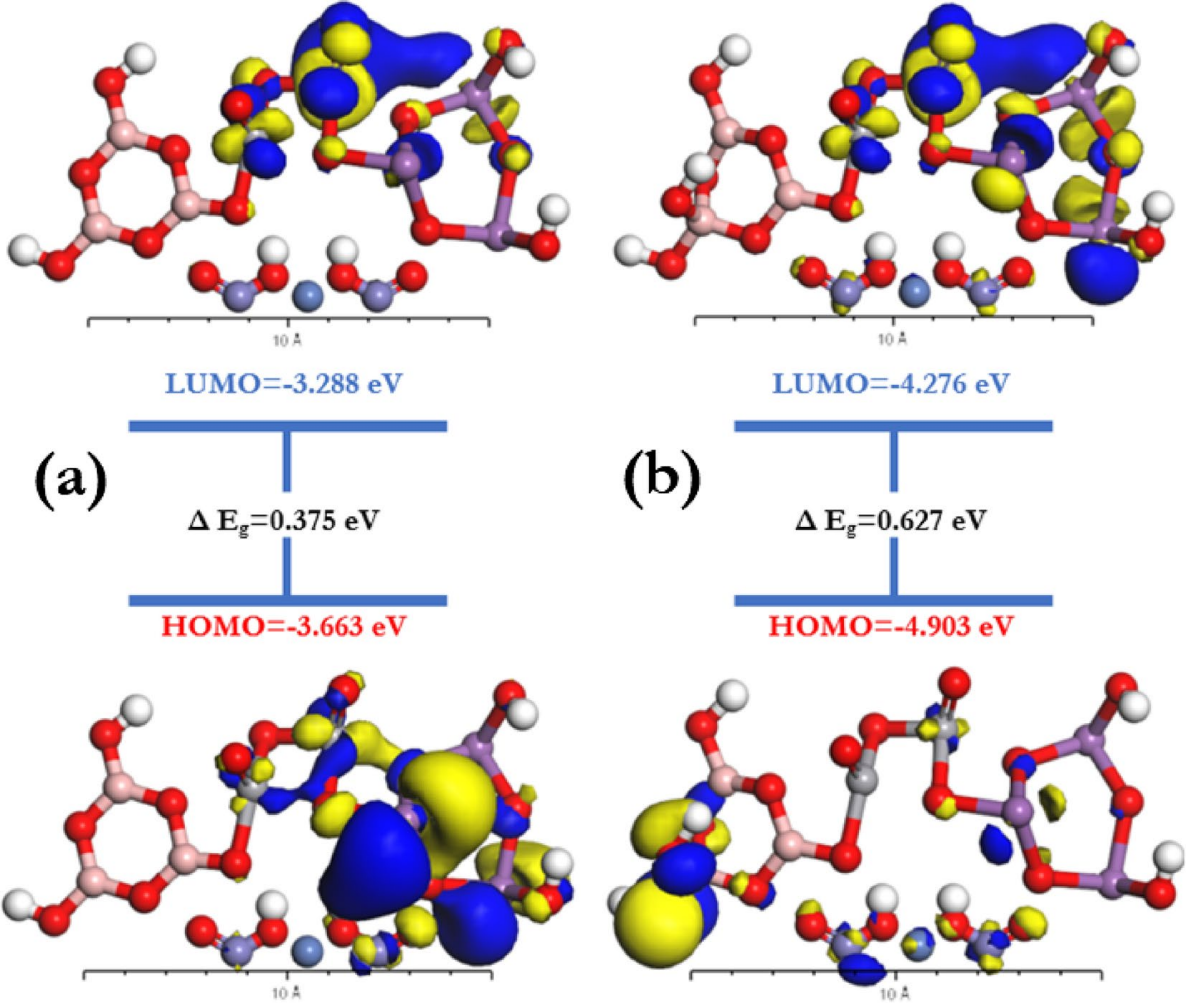
Energy band diagrams of a random [BSV / NiFe_2_O_4_]^Iso^ between the highest occupied molecular orbital (HOMO) and the lowest unoccupied molecular orbital (LUMO).

Depending on the discrepancy between the highest occupied molecular orbital (HOMO) and the lowest unoccupied molecular orbital (LUMO) the optical energy gab E_g_ was measured using DFT-*Dmol*^*3*^ as shown in Fig. 7. Fragment molecular orbitals (FMOs) are directly dependent on the simulations of the HOMO and LUMO states of the molecules. The presence of the 4-coordinate boron atom has a high impact on the energy values of HOMO and LUMO states which results in increasing the energy gap value from 0.375 to 0.627 eV. The reason behind the decrease in HOMO and LUMO energies is the formation of holes in the fourth boron-oxygen bond.

Important physio-chemical parameters Like chemical potential (μ), softness (σ), global softness (*S*), global hardness (η), electronegativity (χ), global electrophilicity index (ω), and the maximum amount of electronic charge (Δ*N*_*max*_) can be easily calculated depending on the values of HOMO and LUMO states energies using the equations of ($\mu =({E}_{HOMO}+{E}_{LUMO})/2),$ ($\upeta ={(E}_{LUMO}-{E}_{HOMO})/2)$, $(\upchi =-\upmu )$, $(S=1/2\upeta )$, ($\omega ={\mu }^{2}/2\upeta$), ( $\upsigma =1/\upeta$ ) and ($\Delta {N}_{max}=-\mu /\eta )$^59,60^. The values of E_HOMO_ and E_LUMO_ and the calculated parameters of (*μ*), ($\upsigma )$, (*S*), ($\upeta )$, (χ), (ω), and (${\Delta N}_{max})$ are tabulated in Table 2. The negative values of E_HOMO_ and E_LUMO_ indicate the stability of [BSV / NiFe_2_O_4_]^Iso^, while the critical quantum chemical feature (ω) describes energy stability when an additional electronic charge is received^61^.

**Table 2:** Geometry constants for [BSV / NiFe_2_O_4_] as isolated molecules.

Compounds	E_HOMO_	E_LUMO_	E_g_	χ (eV)	µ(eV)	η (eV)	S (eV)	ω (eV)	$\Delta {N}_{max}$	$\upsigma$
Boroxol ring	− 3.663	− 3.288	0.375	3.475	− 3.475	0.187	2.673	32.28	18.58	5.347
Modified boroxol ring	− 4.903	− 4.276	0.627	4.589	− 4.589	0.313	1.597	33.64	14.66	3.194

### Optical properties

**Figure 8:**
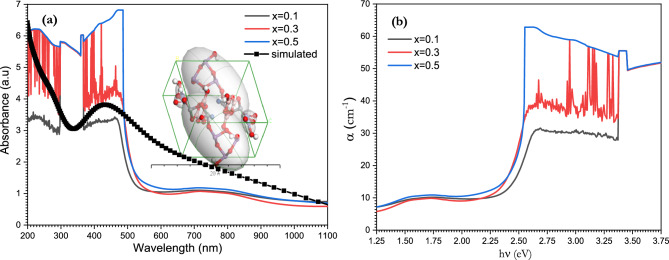
(**a**) UV–visible absorption spectrum, (**b**) optical absorption coefficient of [BSV / NiFe_2_O_4_] glass samples with different concentrations of V_2_O_5_.

The UV–Vis spectra of the [BSV / NiFe_2_O_4_] glass matrix is shown in Fig. 8a. Vanadium is a 3d transition metal and so can exist in three valence states: V^3+^, V^4+^ and V^5+^. The possible absorption band in the figure originates from octahedral coordination with oxygen ions. The intensity of this peak is very low compared to the pure glass BSV glass matrix, which indicates that the presence of ferrite reduced the d-d transition possibility of the electron (this appears at 600 nm). On the other hand, the appearance of this weak broad band is due to the appearance of trivalent vanadium ions in the glass matrix^49^. The absorbance nature of the glass matrix obscured the Fe^3+^ and Ni^2+^ ions absorption bands, which may have taken place at 400 and 450 nm^62–64^.

The absorption coefficient α was calculated in Fig. 8b. From the following equation^65^:

$${\mathrm{\alpha }} \, \left( \lambda \right)\, = \,{2}.{3}0{3}\left( \frac{A}{d} \right)$$

where α is the absorbance and d is the thickness of the material. The absorption coefficient can be increased by increasing V_2_O_5_ content.

**Figure 9:**
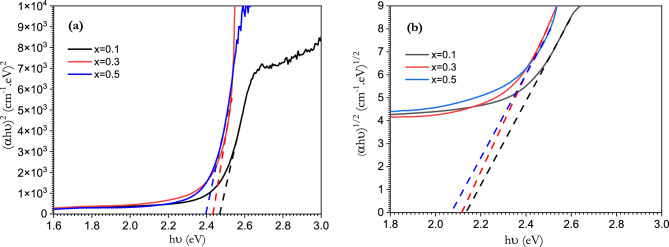
The dependence of (**a**) (αhν)^2^ and (**b**) (αhν)^1/2^ on the photon energy (hν) of [BSV / NiFe_2_O_4_] glass samples with different concentration of V_2_O_5_.

To study the energy difference between the HOMO and LUMO states, the optical energy gab was calculated using Tauc’s relation as^66–68^:

$$\left( {\mathrm{\alpha h \upsilon }} \right)^{{\text{n}}} ={\mathrm{ A (h \upsilon }}- E_{g} {)}$$

where hυ is the energy of the incident photon, A is a constant value and n is an index with a value of 2 for direct allowed transition and 1/2 for indirect transition. The optical band gap was provided from the extrapolation of the linear part of the curve between ${(\mathrm{\alpha h \upsilon })}^{2}$ and ${(\mathrm{\alpha h \upsilon })}^{1/2}$ against hυ as shown in Fig. 9**.** The optical energy gap decreased as increasing V_2_O_5_ content increased, which agrees with energy band theory: this is a result of splitting every state to n, which is equal to the number of interacting atoms. The calculated values of direct and indirect band gap are tabulated in Table 3.

**Table 3:** Value of absorption coefficient (α), energy gap (${E}_{g}$), and the Urbach energy and refractive index of different [BSV / NiFe_2_O_4_] glass samples.

Sample	α_edge_	E_g_^direct^ (eV)	E_g_^indirect^ (eV)	E_U_
[BSV-0.1 V_2_O_5_ / NiFe_2_O_4_]	31.2	2.46	2.13	0.226
[BSV-0.3 V_2_O_5_ / NiFe_2_O_4_]	38.5	2.43	2.11	0.223
[BSV-0.5 V_2_O_5_ / NiFe_2_O_4_]	62.6	2.39	2.06	0.217

**Figure 10:**
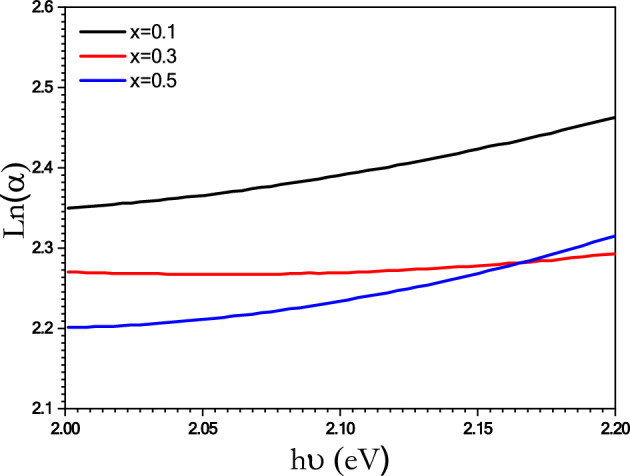
Plot of ln(α) with hν of [BSV / NiFe_2_O_4_] glass samples with different concentration of V_2_O_5_.

The Urbach energy was calculated using the following equation^66,69^:

$${\text{ln }}\alpha \, = \,{\text{ln}}\;(\alpha_{o} ) + \frac{h\upsilon }{{E_{U} }}$$

where, E_U_ was calculated from the reciprocals of the slopes of the linear region of the plot between Ln α and hυ as shown in Fig. 10. The Urbach energy can yield information about disorder effects in amorphous or crystalline systems. It is inverse relation with the energy gap, so it decreases V_2_O_5_ content increases in the presence of nickel ferrite^70^.

**Figure 11:**
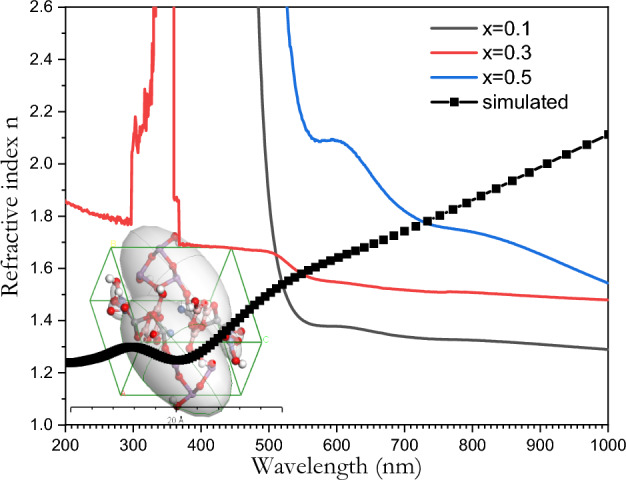
The dependence of the refractive index (n) of the [BSV / NiFe_2_O_4_] glass samples with different concentration of V_2_O_5_ on the wavelength λ(nm).

The refractive index of the [BSV / NiFe_2_O_4_] glass system was calculated according to the following equation^71^:

$${\text{n}}\, = \left( {\frac{1 + R}{{1 - R}}} \right)\, + \left( { \frac{4R}{{\left( {1 - R} \right)^{2} }} - K^{2} } \right)^{{{\raise0.7ex\hbox{$1$} \!\mathord{\left/ {\vphantom {1 2}}\right.\kern-0pt} \!\lower0.7ex\hbox{$2$}}}}$$

where K is the extinction coefficient and R is the reflectance which was determined using the transmittance T and absorbance A as $R=1-\sqrt{T.{e}^{A}}$. The refractive index n was found to increase as V_2_O_5_ content increases, which would make the glass useful in optoelectronic applications. The difference in the behavior between the refractive index and the absorbance spectra results from electromagnetic waves scattering inside the glass sample due to the presence of nickel ferrite particles. The calculated values of the refractive index are shown in Fig. 11.

### Photoluminescence

**Figure 12:**
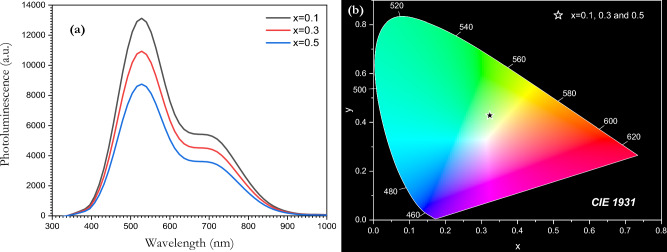
(**a**) Photoluminescence, (**b)** CIE digital photograph of the [BSV / NiFe_2_O_4_] glass samples.

The photoluminescence spectra of [BSV / NiFe_2_O_4_] are shown in Fig. 12a. The main emission peak is located at around 527.81 nm with another peak appearing at 707 nm. The emission intensity decreases as V_2_O_5_ content increases. This exotic behavior is related to the presence of nickel ferrite particles. The photoluminescence of [BSV / NiFe_2_O_4_] was analyzed using Commission Internationale de l'Eclairage (CIE) graphs to illustrate the emission colour of the glass composites (Fig. 12b). The emission colour was affected by the concentration of V_2_O_5_. All the glass samples emit green light which is located at (0.32, 0.43) in the CIE digital photographs. This indicates that the glass can be used for light emitting diodes (LEDs).

From the ${\lambda }_{emission}$ value we calculated the optical energy gap as equal to 2.35 eV from photoluminescence using the following equation^72,73^:

$$E_{g}^{PL} = \frac{1240}{{\lambda_{emission} }}$$

### Laser irradiation of the glass composite:

**Figure 13:**
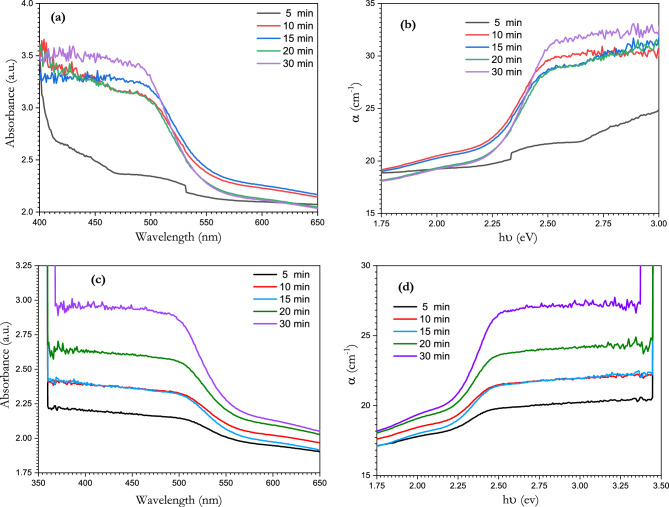
UV–visible absorption spectrum of [BSV-0.1 V_2_O_5_ / NiFe_2_O_4_] glass with different time exposures to (**a**) continuous and (**c**) pulsed laser irradiation. Optical absorption coefficient of [NiFe_2_O_4_/BG/ 0.1V_2_O_5_] glass with different time exposures to (**b**) continuous and (**d**) pulsed laser irradiation.

Using a continuous diode laser with a power of 150 mW and 375 nm wavelength and a nano-pulsed Nd-Yad laser with power of 50 mW and 1064 nm wavelength, [BSV-0.1 V_2_O_5_ / NiFe_2_O_4_] was irradiated at room temperature over different lengths of time (5, 10, 15, 20 and 30 min) to study the effect of continuous and pulsed laser irradiation on the optical properties of borate antimony glass. Figure 13a and c represent the absorption spectra of the borate antimony glass after irradiation by continuous and pulsed lasers, respectively. The irradiation effect is represented in the increased intensity in the absorption band around 500 nm, which also underwent a slight blue shift. The pulsed laser produced a uniform change in the absorbance spectra, unlike the continuous laser. Figure 13b and d represents the absorption coefficient of the borate antimony glass after irradiation using continuous and pulsed lasers, respectively.

**Figure 14:**
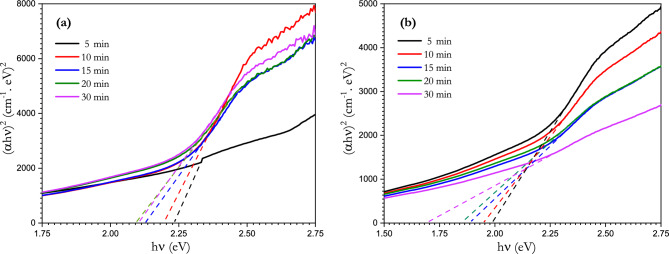
The dependence of (αhν)^2^ on photon energy (hν) of the [BSV-0.1 V_2_O_5_ / NiFe_2_O_4_] glass samples exposed for (**a**) continuous and (**b**) pulsed laser irradiation at different time intervals.

Direct optical energy gaps were calculated for the irradiated glass sample at different irradiation times using Tauc’s relation: this is represented in Fig. 14a and b for the continuous and pulsed lasers, respectively. The pulsed laser reduced the values of the energy gap more than the continuous laser and with much uniform behavior. The energy gap was reduced from 2.397 eV before irradiation to 2.10 and 1.69 eV after 30 min of irradiation using the continuous and pulsed lasers, respectively. Broadening the valence band occurs mostly in the top of the valence band which is formed mainly from ion/ electron pairs. Exposure to laser irradiation leads to the excitement of the electrons at a higher energy level above the top of valence band. However, it does not reach the conduction band, which leads to a broadening of the valence band.

**Figure 15:**
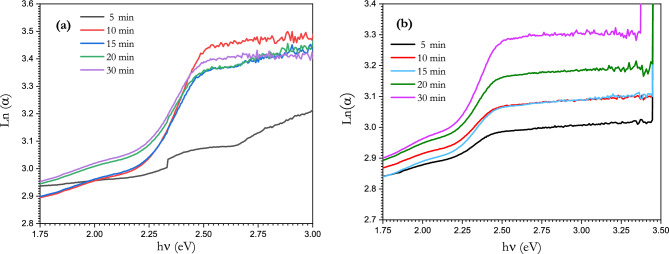
Plot of ln(α) with hν of [BSV-0.1 V_2_O_5_ / NiFe_2_O_4_] glass samples exposed to (**a**) continuous and (**b**) pulsed laser irradiation at different time intervals.

The Urbach energy was calculated for the irradiated samples from the relationship between Ln (α) and photon energy (Fig. 15a and b) for the continuous and pulsed lasers. The Urbach energy was also found to be affected by irradiation, increasing from 0.226 eV before irradiation to 0.72 and 3.57 eV after 30 min of irradiation for the continuous and pulsed lasers, respectively. The changes in the values of the energy gap and Urbach energy after laser irradiation indicate the irradiation effect on the structure and disorder of the glass sample. The calculated values of the absorption coefficient, energy gap and Urbach energy are shown in Table 4.

**Table 4:** Value of absorption coefficient (α), energy gap (${E}_{g}$) and Urbach energy for [BSV-0.1 V_2_O_5_ / NiFe_2_O_4_] glass samples with different time exposures to continuous and pulsed laser irradiation.

Time (min)	E_g_ (eV)		E_U_ (eV)	
	Continuous	Pulsed	Continuous	Pulsed
0	2.397	0.226		
5	2.32	1.98	0.25	1.09
10	2.19	1.95	0.52	1.25
15	2.12	1.88	0.64	1.89
20	2.09	1.84	0.72	2.86
30	2.10	1.69	0.72	3.57

## Conclusion

In this work, a [BSV / NiFe_2_O_4_] glass system was prepared. XRD analysis confirms the system’s amorphous nature, even in the presence of nickel ferrite in the cubic spinel structure. Using UV spectra to calculate the direct and indirect band gaps, their values were lower than pure glass: their decline as vanadium oxide content increased indicates that in the presence of ferrite, the d-d transition of the electron is weak. The study of [BSV / NiFe_2_O_4_] shows that α and (E_g_) increased, as the presence of ferrite decreases the number of non-bridging oxygen ions. The photoluminescence of [BSV / NiFe_2_O_4_] shows the ${\lambda }_{emission}$ of different samples due to spontaneous emission and shows no shift in their values. The study of different thermal properties (DSC) was used to detect the thermal stability of different samples. This shows that [BSV / NiFe_2_O_4_] is more stable than pure glass. The effect of laser irradiation using a diode continuous laser (375 nm wavelength and 150mWpower and pulsed laser 1064 nm wavelength and 50mW power nanosecond pulse duration) was studied for the [BSV-0.1 V_2_O_5_ / NiFe_2_O_4_] glass sample. Laser irradiation shifts the absorbance to a higher value, while α increased as time passed. The value of energy gap decreased with time, while Urbach energy increased: the continuous and pulsed laser n narrowed the optical band gap.

## Data Availability

All data generated or analyzed during this study are included in this published article.
